# Aryl hydrocarbon receptor: The master regulator of immune responses in allergic diseases

**DOI:** 10.3389/fimmu.2022.1057555

**Published:** 2022-12-19

**Authors:** Farooq Riaz, Fan Pan, Ping Wei

**Affiliations:** ^1^ Shenzhen Institutes of Advanced Technology, Chinese Academy of Sciences (CAS), Shenzhen, China; ^2^ Department of Otolaryngology, Children’s Hospital of Chongqing Medical University, National Clinical Research Center for Child Health and Disorders, Ministry of Education Key Laboratory of Child Development and Disorders, Chongqing Key Laboratory of Translational Medical Research in Cognitive Development and Learning and Memory Disorders, Chongqing, China

**Keywords:** aryl hydrocarbon receptor (AhR), allergy, innate immunity, adaptive immunity, T cell, autoimmune disease

## Abstract

The aryl hydrocarbon receptor (AhR) is a widely studied ligand-activated cytosolic transcriptional factor that has been associated with the initiation and progression of various diseases, including autoimmune diseases, cancers, metabolic syndromes, and allergies. Generally, AhR responds and binds to environmental toxins/ligands, dietary ligands, and allergens to regulate toxicological, biological, cellular responses. In a canonical signaling manner, activation of AhR is responsible for the increase in cytochrome P450 enzymes which help individuals to degrade and metabolize these environmental toxins and ligands. However, canonical signaling cannot be applied to all the effects mediated by AhR. Recent findings indicate that activation of AhR signaling also interacts with some non-canonical factors like Kruppel-like-factor-6 (KLF6) or estrogen-receptor-alpha (Erα) to affect the expression of downstream genes. Meanwhile, enormous research has been conducted to evaluate the effect of AhR signaling on innate and adaptive immunity. It has been shown that AhR exerts numerous effects on mast cells, B cells, macrophages, antigen-presenting cells (APCs), Th1/Th2 cell balance, Th17, and regulatory T cells, thus, playing a significant role in allergens-induced diseases. This review discussed how AhR mediates immune responses in allergic diseases. Meanwhile, we believe that understanding the role of AhR in immune responses will enhance our knowledge of AhR-mediated immune regulation in allergic diseases. Also, it will help researchers to understand the role of AhR in regulating immune responses in autoimmune diseases, cancers, metabolic syndromes, and infectious diseases.

## Introduction

The aryl hydrocarbon receptor (AhR) belongs to the PAS (Per-ARNT-Sim), a superfamily of transcriptional factors widely found in numerous tissues throughout the body (1, 2). Genes belonging to PAS domains, typically AhR, enhance the environmental adaptations by sensing and detecting the environmental signals, including the redox potential or oxygen tension (HIF-1α, -2α, and -3α) and circadian rhythm (BMAL-1 and -2) (3). Besides, numerous chemicals and toxins, such as polycyclic aromatic hydrocarbons (PAHs) and Polyhalogenated aromatic hydrocarbons (PHAHs), affect the AhR-dependent responses. Among these, 2,3,7,8- tetrachlorodibenzo-[p]-dioxin (TCDD) is a PHAH toxin that is the most potent ligand for AhR (4). Upon activation, AhR alters the immunotoxicological outcomes and the transcription of AhR-targeted genes cytochrome P450 1A1 and cytochrome P450 1B1 (5, 6).

It was discussed that, upon activation, AhR regulates the transcription of multiple genes by translocating from the cytoplasm to the nucleus. However, recent studies imply that numerous physiologic ligands, e.g., intestinal microbiota, metabolites, and diet, interact with the host to influence AhR-dependent transcription. Identification and characterization of these natural ligands in AhR transgenic mice have demonstrated their pivotal role in AhR signaling (7).

As a transcriptional factor, AhR cooperates with various environmental factors, which act as agonists for AhR, and participates in the disease progression (7–9). AhR sensing and responding to environmental stimuli can play fundamental roles in cellular developmental processes and immune regulation (10). It has been well established that AhR plays a modulatory role in mediating the innate and adaptive immune response to combat various infectious diseases (11, 12), metabolic diseases (13–15), cancer (16, 17), and allergic diseases (18, 19). In the existing manuscript, we will review the role of AhR on the immune cells in response to environmental allergens.

## Mode of action of AhR

**Figure 1:**
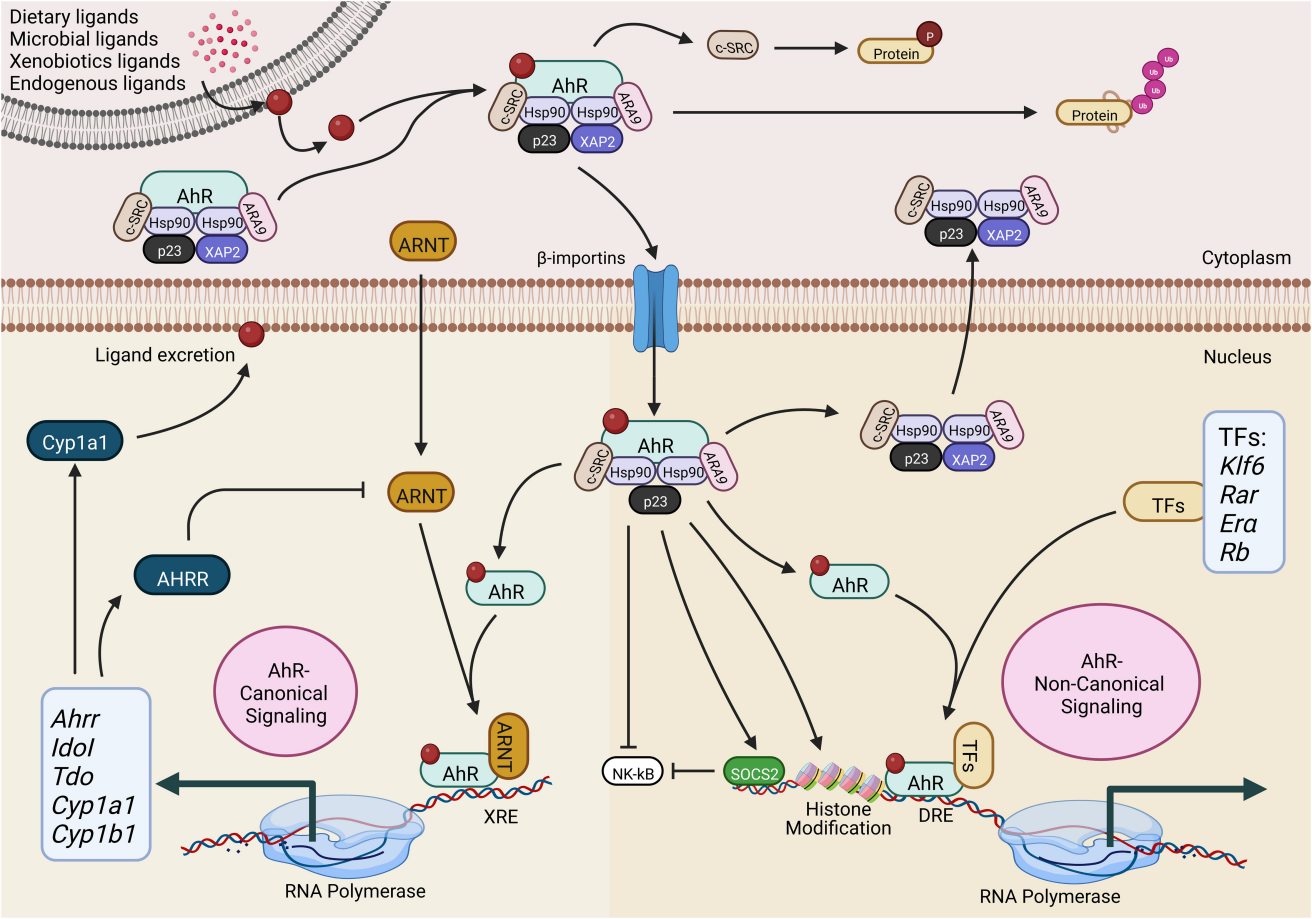
Activation of the aryl hydrocarbon receptor (AhR) canonical and non-canonical signaling pathway. AhR is present in all cell types in the form of a complex with Hsp90, ARA9, XAP2, p23 and c-SRC. Upon interaction with exogenous or endogenous ligands, AhR-complex undergoes conformational changes and uses importin β for the nucleocytoplasmic shuttling. In the AhR-canonical signaling pathway, AhR interacts with ARNT and binds to the XRE to regulate gene expression. In the AhR-non-canonical signaling pathway, AhR binds to various other transcription factors to regulate gene expression. AhR-aryl hydrocarbon receptor; ARNT-AhR nuclear translocator; AhRR-AhR repressor; CYP1A1, cytochrome P450 1A1; CYP1B1-cytochrome P450 1B1; c-SRC-proto-oncogene tyrosine-protein kinase Src; ARA9-immunophilin homolog ARA9; HSP90-heat shock protein of 90 kDa; p23-prostaglandin E synthase 3; XAP2-hepatitis B virus X-associated protein 2; IDOI-indoleamine 2,3-dioxygenase 1; TDO-tryptophan 2,3-dioxygenase; TF-transcription factor; KLF6-Krüppel-like factor 6; RAR-retinoic acid receptor; ERα-oestrogen receptor-alpha; Rb-retinoblastoma protein; SOCS2-suppressor of cytokine signaling 2; XRE-xenobiotic response element; DRE- Ub-ubiquitylation; P-phosphorylation. The illustration is drawn by BioRender (biorender.com).

AhR, a well-conserved protein, is ubiquitously expressed in mammalian organs with flexible expression levels amongst different tissues at different stages of life (20–22). Briefly, in an inactive state, AhR is present within the cell cytosol in the form of a protein complex. During activation after binding to its ligands, such as an allergen or environmental toxin, AhR undergoes conformational changes and translocates to the nucleus. After translocation, AhR triggers the transcription of numerous target genes through dioxin-responsive elements (DREs) (23, 24). Conversely, AhR prevents self-activation in a negative feedback loop by degrading AhR through AhR repressor (AhRR), which is a primary downstream target of AhR (23, 24). In addition to this canonical process, AhR also influences biological processes in a non-canonical way. In a non-canonical pathway, the cytoplasmic AhR complex is dissociated from its ligand-receptor complex. It results in the release of many biologically active molecules, including the c-SRC kinase, which leads to the phosphorylation of numerous genes (25). Remarkably, ligand-AhR acts as a facilitating protein during the formation of the ubiquitin ligase complex, which eases the degradation of steroid receptors, e.g., the central regulator of adipogenesis peroxisome proliferator-activated receptor γ (PPARγ) (26) or estrogen receptor (Erα) (7, 27), by enhancing the substrate specificity. The AhR-canonical and non-canonical signaling pathway is illustrated in **Figure 1**.

Before exposure to the ligand in the cytoplasm, inactive AhR is present in the form of a protein complex that is comprised of the hepatitis B virus X-associated protein (XAP2), the chaperone heat-shock protein 90 (HSP-90), the co-chaperone p23 (p23), and the c-SRC protein kinase (21, 25, 28–31). Hsp90 protein keeps the right conformation of AhR for receptor bindings but also averts the AhR translocation to the nucleus (32). Conversely, the co-chaperone p23 is highly involved in stabilizing the interaction and complex of AhR-Hsp90, while the ARA9 elevates the AhR activation by properly folding AHR in the cytoplasm (33, 34).

Furthermore, HSP90 adjusts the AhR in the correct position to provide a high affinity for its ligands (32), while AhR steady-state cellular levels are maintained by AIP, which prevents AhR ubiquitination and degradation (35). The release of AIP from the complex exposes the nuclear signals of AhR. This leads to pivotal conformational changes in AhR; thus, it may lead to the nuclear translocation of AhR (36). On the other side, the nuclear translocation of AhR is also dependent on the phosphorylation of protein kinase C (37), establishing the fact that AhR is being controlled through several mechanisms. Additionally, regulating the nuclear translocation of AhR can be a potential therapeutic target for the precise reorientation of non-canonical AhR signaling.

During the activation of the canonical AhR signaling pathway in the presence of agonists, the ARA9-AhR-HSP90-p23 complex undergoes a conformational change which helps the translocation of this complex to the nucleus *via* β-importins (21, 25, 28–31). Several investigations suggest that XAP2 anchors the AhR complex in the cytoplasm. Other inquiries contend that XAP2 restricts the interaction of β-importins with nuclear localization signal. Importantly, prior to the nucleocytoplasmic shuttling, the release of XAP2 from the AhR complex is necessary (36, 38–40).

Further studies conducted in HeLa cells indicated that translocation of AhR to the nucleus could happen without being dissociated from HSP90 (38). Though, it’s unclear whether this finding can be generalized to various cell types or other AhR agonists (41, 42). Technically, AhR within the nucleus undergoes conformational changes by heterodimerizing with the AhR nuclear translocator (ARNT). Meanwhile, it also binds with particular DNA sequences, known as xenobiotics- or dioxin-response elements (XRE or DRE), to modulate the level of specific genes (29, 43–46). These genes contain numerous enzymes involved in xenobiotic metabolization, e.g., NAD(P)H-quinone oxidoreductase, CYP1A1, CYP1A2, and CYP1B1 (47).

In addition, AhR can control the expression of various genes directly by interacting with specific DNA sequences or indirectly by interacting with regulatory RNAs and transcription factors. These AhR-mediated regulations of gene expressions are generally achieved in an AhR-non-canonical signaling pathway manner. Practically, 5’-TNGCGTG-3’ is a DNA consensus motif located in the AhR target gene responsible for the interaction of AhR in the genomic regulatory regions. These DNA consensus motifs are recognized as DRE or XRE (48). Besides interacting with DREs or XREs, the AhR/ARNT complex also modulates the expression of various genes by interacting with various transcriptional factors. AhR exerts a significant effect during the chromatin remodeling during the interaction of AhR with chromatin-remodeling complexes, i.e., the steroid receptor coactivator-1 (SRC-1) (49), SWI/SNF (50), and by relocating the histone deacetylase (HDAC) complexes (51).

AhR has also been categorized to regulate the expression of a variety of downstream target genes by its interaction with their respective transcription factors, e.g., retinoblastoma protein (Rb), the estrogen receptor and E2F, the retinoic acid receptor, c-Maf, and NF-κB (52). AhR-dependent activity of these transcription factors is involved in mediating the numerous feedback loops which are extremely related to immune regulation. For instance, NF-κB elevates the AhR expression; however, AhR can alternatively modulate the NF-κB signaling (53–55).

Similarly, an alternative to canonical DREs, AhR can also be associated with numerous other transcription factors, which can help AhR to be directly recruited to the target DNA sequences. For instance, dimerization of AhR with RelA and RelB can direct the recruitment of AhR at the responding sites of NF-κB sites (56, 57) and with KLF6 (58). It suggests that non-canonical AhR signaling leads to the AhR recruitment to various non-consensus DNA elements. Strikingly, various ligands have been associated with enhancing the interaction of AhR with multiple genes (59). The recruitment of AhR at different target sites can subsequently alter the biological and cellular processes, including the immune responses in allergic diseases, cancers, and autoimmune diseases.

## Allergic diseases

It is estimated that nearly 22% of the global population suffers from allergic disorders. These include eczema, allergic asthma, allergic rhinitis, drug or food allergic reactions, anaphylaxis, and allergic conjunctivitis (60, 61). Globally, allergic diseases are touching epidemic proportions with a particular increase among westerners. Since allergic diseases are highly associated with inflammation, various studies suggest a close association of allergic diseases with the onset of numerous complicated diseases, particularly metabolic diseases (62), cancer (63), mental disorders (64), stroke (65), and cardiovascular diseases (66). Similarly, allergic rhinitis and asthma are related to the high risk of obesity, insulin resistance, and blood sugar (67–70). These studies suggest that the pathogenesis of allergic diseases also can serve as an etiological factor for various other diseases. Therefore, we must improve our knowledge about allergic diseases and the impact of these allergic diseases on other associated diseases (71).

The current description of allergic diseases as abnormal conditions principally initiated in an immunoglobulin E (IgE)-dependent mechanisms is not well-applicable to all allergy patients (72). Typically, in allergic individuals, binding of IgE with primary allergens triggers allergic responses in ~50% of patients. In comparison, other non-primary allergens trigger an allergic reaction and activate various immune cells (73, 74). Therefore, it is necessary to determine the appropriate functions of immune cells in allergic inflammatory responses, so we can develop innovative, beneficial strategies to constrain allergic diseases.

## Molecular mechanism of allergic diseases

**Figure 2:**
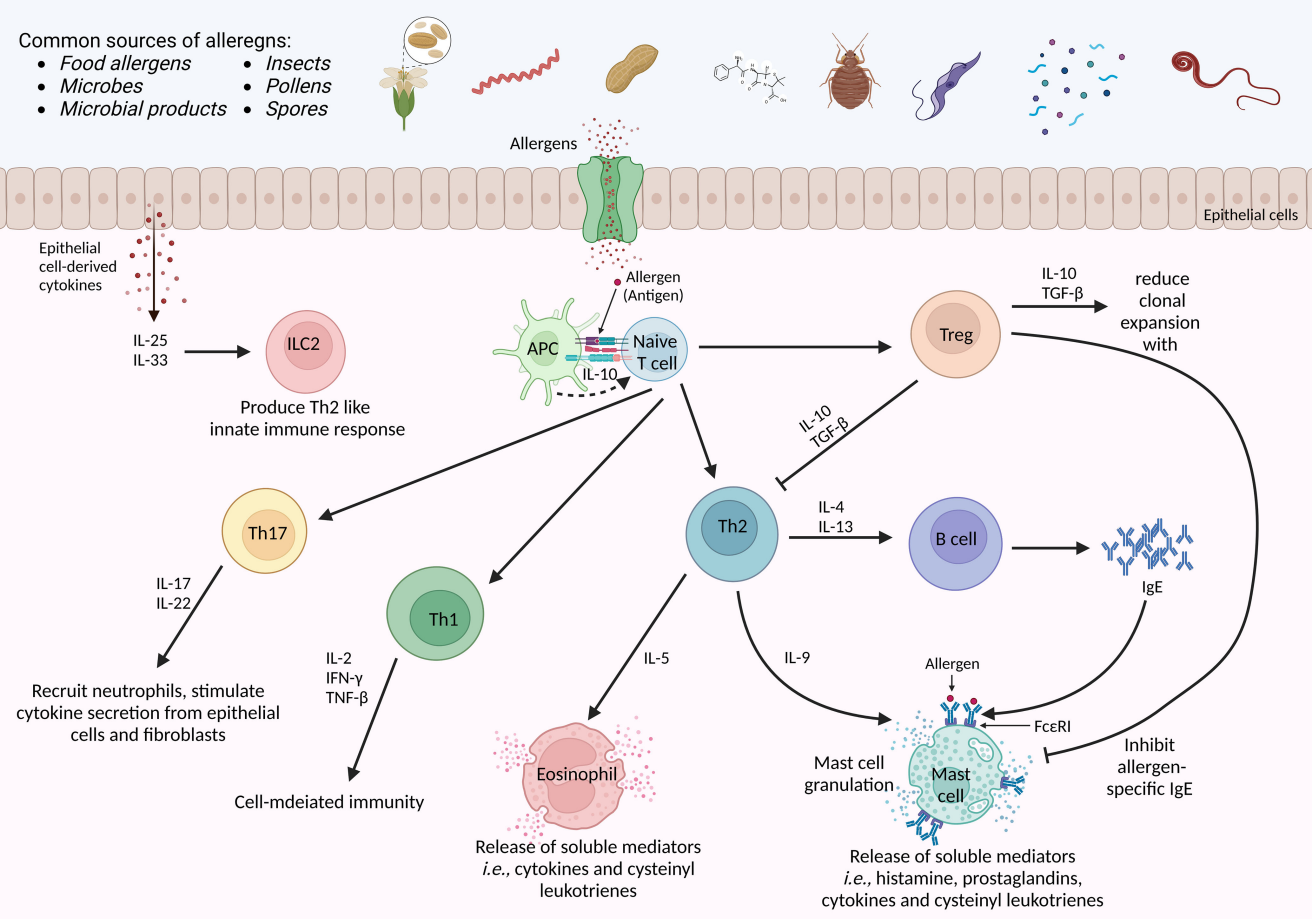
Molecular mechanism of an allergens-induced immune response. The host is exposed to multiple sources of allergens daily. Allergens-induced immune response begins when an allergen is presented to naïve T cells *via* antigen-presenting cells. Upon activation in the presence of first allergen exposure, naïve T cell is differentiated into Th2 cells, and the production of IgE by B cells. The IL-10 from APC favors the selective clonal expansion of Th2 cells, particularly the Th2 memory cells. in case of allergen re-exposure, crosslinking of antibody-loaded high-affinity IgE receptors is triggered on the surface of mast cells to provoke an acute-phase immune response. Subsequently, several pro-inflammatory mediators are secreted by Th2 cells, eosinophils, and mast cells in a late-phase reaction. Besides, Tregs suppress Th2-cell responses by secreting IL-10 and TGF-β. Meanwhile, Th1 secretes various cytokines to achieve cell-mediated immune responses, while Th17 recruits neutrophils and triggers epithelial cells and fibroblasts to secrete cytokines. IFNγ-interferon-γ, FceRI, the high-affinity receptor for IgE; TGF-β-transforming growth factor-beta; APC-antigen presenting cell; IL-interleukin. The illustration is drawn by BioRender (biorender.com).

Significant development has been made in understanding the intrinsic and structural biology of potential allergens and environmental agents (75). Allergic inflammatory reactions are specified through the assortment of the Th2-cell-dependent signaling pathway. This Th2-cell pathway begins upon the exposure of allergens and their uptake by the first line of defense antigen-presenting cells (APCs). APCs display the particular peptides/antigens to naïve T cells *via* MHC class II receptor. Thereby. Naïve T cells are directed towards the Th2-cell phenotype, which generally mediates the expression of secretory cytokine *via* transcription factor GATA3 (GATA-binding protein 3) (76). Meanwhile, Th1 phenotypic responses are generalized by the secretion of cytokines, i.e., interferon-γ (IFNγ) through the T-bet transcriptional factor. Overall, T cells play a leading role during the development and advancement of autoimmune, allergic, and cancerous diseases (**Figure 2**) (77).

Besides, it has been characterized that IgE binds to mast cells to activate mast cell degranulation and contributes to presenting the antigen to other immune cells. On the contrary, eosinophils yield some pro-inflammatory cytokines that can activate bronchial hyperreactivity and allergic inflammation (78, 79). Meanwhile, when APCs present potential antigens to naïve T cells *via* MHC-II, T cells are activated. Activated T cells coordinately elevate the secretion of a bunch of cytokines encoded by human chromosome 5q31–33. Briefly, activation of T cells encodes granulocyte/macrophage colony-stimulating factor (GM-CSF) and interleukin-3 (IL-3), IL-4, IL-5, IL-9, and IL-13 (80). These cytokines play crucial roles during allergic inflammation.

The Th2/Th1 imbalance during allergic responses has been established for more than two decades (81). However, recent investigations have discovered regulatory T (Tregs) cells as an additional crucial subset of CD4^+^ T cells, which is essential in mediating allergic disease (82). Increasing evidence from *in-vivo* allergic mice models sturdily associate Tregs with the suppression of allergen-specific responses and allergic inflammation (83). Moreover, numerous studies also stated that Tregs regulate the Th2-cell-mediated allergic responses by secreting various suppressive cytokines, including transforming growth factor-β (TGFβ) and IL-10, and urged that allergic reactions are consequent of Th2/Treg imbalance (84).

As the prevalence and morbidity rate of allergies is growing worldwide (85, 86), it is necessary to develop and design new treatment strategies that mask the symptoms of allergies and reduce the basic allergic cascade during allergic inflammation. As mentioned earlier, immune cell-mediated allergic responses are gaining importance in the scientific community. However, how immune cells mediate the immune responses to counter environmental nonpathogenic antigens, which progress to allergic reactions, is not well understood.

## AhR in regulating immune responses in allergic diseases

Striking evidence indicates the extensive role of IgE and eosinophils in the pathogenesis of allergic inflammation and the development of therapeutic strategies targeting IgE and eosinophils (87). Nevertheless, the role of other immune cells in allergic reactions hasn’t been investigated well. Meanwhile, the role of AhR in regulating the inflammatory effects of environmental pollutants and allergens makes it an attractive target for achieving therapy against allergic diseases (88). In response to potential allergens and AhR ligands, the expression of AhR and the downstream signaling pathway exert various roles in different immune cells (**Table 1**). Therefore, it exhibits pleiotropic properties by participating and integrating with other signaling pathways, i.e., the Wnt-β catenin network and sex hormones receptors (101). The preliminary development of AhR^-/-^ mice showed that AhR deficiency considerably reduced the lifespan, as indicated by frequent death after birth. Moreover, in the first few weeks of birth, AhR^-/-^ mice also represented a compromised growth rate (102, 103). In addition, AhR^-/-^ mice developed various hepatic abnormalities, such as abnormal retinoic acid metabolism, transient hepatic steatosis, and bile duct fibrosis (102–104). Similarly, an *in-vivo* model of imiquimod-induced psoriasis exhibits that, instead of hematopoietic cells, AHR in keratinocytes limits inflammation (105). Besides, an indole derivative IAId of microbiota-derived tryptophan metabolite exerts inverse effects on skin inflammation in atopic dermatitis patients by targeting AhR (106). It is known that AhR-deficient animals display high resistance to TCDD, which is an environmental pollutant. However, an in-depth examination of the immune responses was lacking (107). AhR regulates allergic diseases in both canonical and non-canonical pathways. It has been found that Di(2-ethylhexyl) phthalate (DEHP), a plasticizer, boosts the ovalbumin-induced allergic rhinitis by activating the AhR canonical pathway (108). However, a detailed understanding of the impact of the AhR-canonical signaling pathway on the AhR-non-canonical signaling pathway hasn’t been studied.

**Table 1:** Role of potential allergens and AhR ligands on the immune responses in allergic diseases.

Potential allergens/AhR ligands	Model	Response	References
TCDD	Non-eosinophilic asthma model	Increased Th17 and decreased Treg differentiation	(89)
FICZ	Mouse bone marrow-derived mast cells from AhR^-/-^ mice	Maturation and activation of mast cells, and secretion of IL-6 and IL-17 by mast cells	(90, 91)
Indoxyl 3-sulfate (I3S)	Ovalbumin-sensitized allergic asthma	Regulate Th2 differentiation	(92)
Ovalbumin aerosol	Ovalbumin allergy model	T cell activation by dendritic cells	(93)
4-nonylphenol Indeno[1,2,3-cd]pyrene	Ovalbumin-induced allergic asthma	Increased secretion of Th2 cytokines	(94, 95)
Cockroach extract (CRE B46)	Allergic asthma	Polarization of M1/M2 macrophages	(96)
Urban dust particle (SRM1649b)		Enhance Th17 polarization	(97)
PM2.5	Cockroach-sensitized mouse model	Increased Th17 and decreased Treg differentiation	(98)
Non-dioxin-like AhR ligands (FICZ, β-NF and 6-MCDF)	Peanut allergy model	No effect on Treg	(99)
Indole-3-carbinol	Peanut allergy model	Increase in CD11c+ and CD103+MHC-II+ cells	(100)

**Figure 3:**
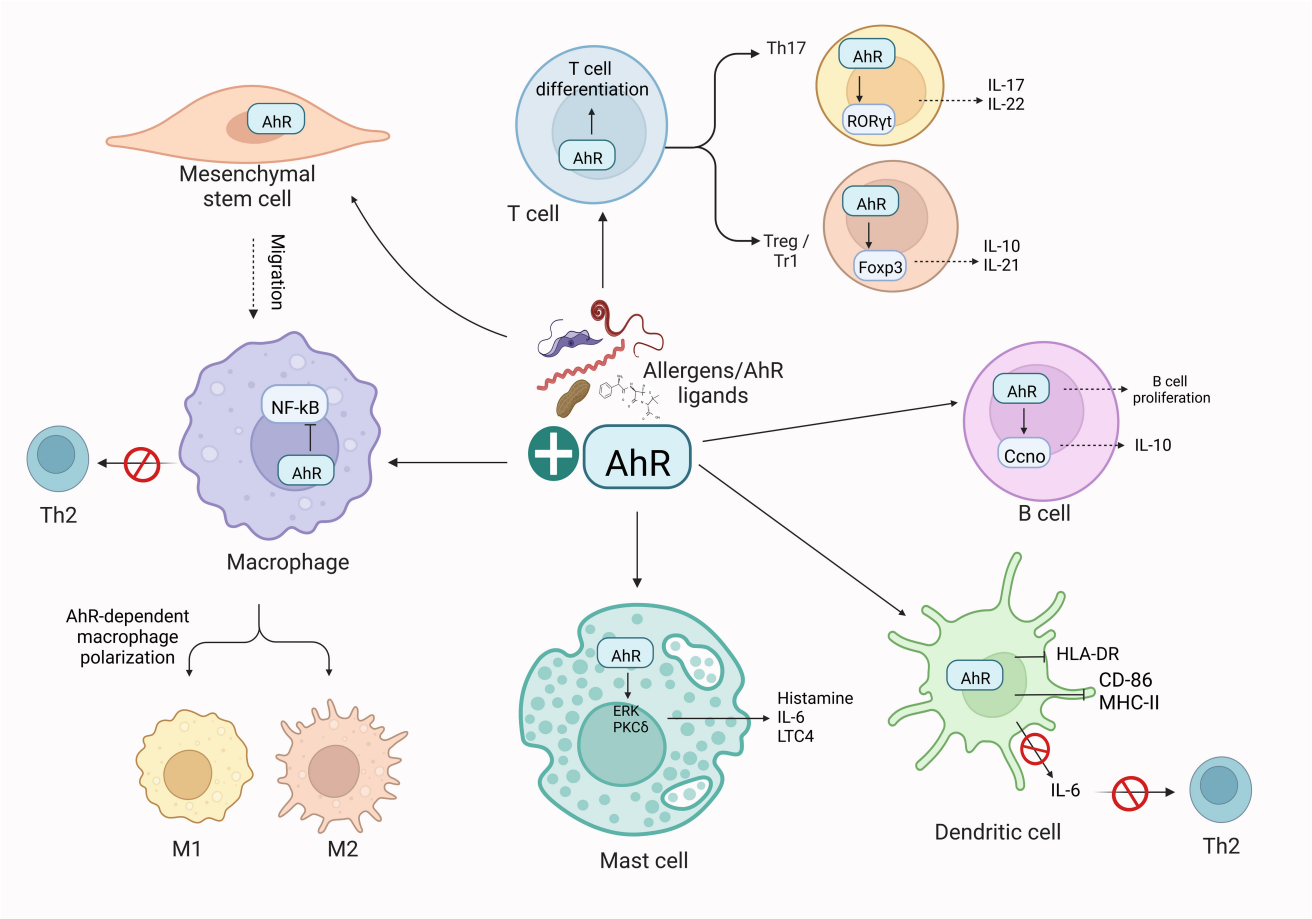
AhR-dependent regulation of immune cells. Activation of AhR in response to exogenous ligands or allergens influences the activity of adaptive and innate immune responses. Briefly, AhR affects the polarization of macrophages, the function of Th2 cells, T and B cell differentiation, and DC functions. These effects of AhR ultimately imbalance the M1/M2 polarization and Th17/Treg balance. The illustration is drawn by BioRender (biorender.com).

Emerging investigations have specified the profound influence of AhR signaling on host-pathogen and host-commensal interactions and in shaping the immune responses from various cell types. AhR is a critical player in mucosal interfaces where an individual interacts with environmental pollutants and other living entities (109, 110). As stated, the AhR might serve a considerably efficient role in mediating allergic immune responses by acting as an essential factor in regulating the functions of various immune system cells, together with B, T, and dendritic cells (18, 23, 111, 112). Briefly, AhR mediates mast cell differentiation and growth (90, 113), dendritic cell function (114), differentiation of type 1 regulatory T cell (Tr1) (115) and Th9 (116), intraepithelial lymphocytes functions (117), Treg intestinal homing (118) and impacts on γδ+ T cells homeostasis (119), affects the balance of Tregs and Th17 cells (120, 121), and lymphoid follicles (122). A brief overview of the role of AhR in immune cells has been shown in **Figure 3**. These findings suggest that AhR is critical for the progression of allergic inflammatory diseases and the development of allergic and inflammatory responses. However, the detailed mechanism of the impact of AhR in allergen-induced allergic inflammation and underlying mechanisms persist indefinably.

## AhR in mast cells to regulate allergic diseases

As discussed earlier, mast cells are critical to exert adjuvant functions during exposure to allergens and immune sensitization in response to these allergens (123, 124). Generally, mast cell produces a broad variety of inflammatory factors to respond to environmental allergens and initiate immune responses (125). Activation of rat RBL2H3 mast cell line with AhR endogenous ligands, kynurenine and kynurenic acid, elevates the level of IL-6, a well-known AhR downstream target, in a calcium-dependent manner (126). Later, the expression of AhR was reported in the rat RBL2H3 mast cell line (127) and the mouse and human mast cells (90, 91). Afterward, several lines of evidence indicate the role of AhR in the immune regulation of mast cells. Ligation of AhR with its ligand 6-formylindolo-[3,2-b]-carbazole (FICZ) potentiate the classical IgE/Ag-dependent response by elevating the secretion of pro-inflammatory cytokines IL-6 and IL-13, LTC4 and histamine, through the activation of PKCδ and ERK (90, 91). On the other hand, AhR activation also induces the production of reactive oxygen species (ROS) in response to AhR activation through mast cells to impact mast cell-dependent inflammatory responses (128). In association with this, chronic rhinosinusitis with nasal polyps (CRSwNP) was found to be associated with mast cell-dependent oxidant stress and inflammation. Mast cell-specific absence of AhR, predominantly found in the mast cells of nasal polyps, reduced ROS production and the expression of oxidized calmodulin-dependent protein kinase II (ox-CaMKII). This suggests the role of AhR in regulating the activation of mast cell by altering the levels of ox-CaMKII and ROS in CRSwNP patients (129). Meanwhile, genetic ablation of AhR in mast cells impairs the production of IL-13 and elevates mitochondrial damage; thus, it participates in the homeostasis and maturation of mast cells (90).

Interestingly, the response of mast cells is highly dependent on the duration of FICZ exposure. It was noted that a single dose of FICZ improved the activation characteristics of mast cells in terms of secretion of pro-inflammatory IL-6 and histamine. In contrast, extended exposure to FICZ boosted IL-17 production and decreased degranulation (91). These IL17+ mast cells were also found in the lung parenchyma of patients with chronic obstructive pulmonary disease (COPD) (91). ORM1 (yeast)-like protein 3 (ORMDL3), localized in the endoplasmic reticulum (ER), is pivotal in allergic asthma (130). A recent study illustrates that AhR ligand, PAH, activates sphingosine-1-phosphate S1P production, and decreases the activity of S1P lyase (S1PL) in resting immune cells, IgE/antigen-activated mast cells. This decreased activity of S1PL is reversed by silencing ORMDL3 or adding an antioxidant, thereby suggesting that the AhR-ligand axis regulates the time-dependent upregulation of ORMDL3/S1PL complex, particularly in allergic diseases (131). Meanwhile, cow/goat milk containing a protein family of lipocalins or secretoglobins acts as potential respiratory allergens (132). In another research, beta-lactoglobulin (BLG), a bovine lipocalin in milk, acts as an allergen and primes human mast cells for degranulation by facilitating the quercetin-dependent AhR activation and influencing the expression of AhR-downstream Cyp1A1 in the lung (133). Conclusively, increasing data reveal the fundamental involvement of mast cells in normal and allergic disease conditions. Mast cells affect the host in allergic diseases and participate in the deregulatory immune response.

## AhR in dendritic cells to regulate allergic diseases

In response to allergens, DCs are crucial in inducing allergic sensitization or tolerance (134). As DCs are present and dispersed across the body, their localization is firmly expressed by their functions as APCs. Immature DCs exist in the place where the entry of the first antigen is projected, such as the urogenital system, upper and lower airways, and the epithelium of skin and gut mucosa. In the same way as other antigens, allergens cross cellular barriers to interact with DCs (134). These cells move to lymphoid organs and transmit antigens to immature T cells. Either the stimulation of Foxp3+ regulatory T (Treg) cells or the induction of T helper 2 (Th2) cells would cause allergic sensitization (135).

Skin-resident specialized DCs can swiftly detect and sample antigens to determine whether they constitute a health risk. Langerhans cells (LCs) are dendritic cells found in the skin. Epidermal LCs were identified as DCs when C3, Fc, and MHC Class II receptors were found on them, and they have been the subject of in-depth research ever since (136, 137). In the mammalian dermis, however, new DC subpopulations have also been found (138).

The level of many co-stimulatory molecules, including CD54, CD80, and CD86, indicates that epidermal LCs interact with allergens as soon as they penetrate the skin barrier and get activated. The secretion of pro-inflammatory cytokines, such as IL-1, IL-12, and TNF-α is also stimulated concurrently (139). According to animal studies, this inflammatory process is followed by the infiltration and buildup of moDC in tissues. The murine DC markers (CD11c, CD80, CD86, MHC II, and DEC205) as well as the macrophage-associated markers (Mac-3, F4/80), and monocyte (CD11b, Ly6C) are expressed by these cells (140).

CD1a+CD11b+CD1c+ plasmacytoid and myeloid DCs have been seen in the skin of people with atopic dermatitis (141). FcR1, a highly affinitive IgE receptor, is expressed by both groups (142–144). Because oral sensitivity to peanuts is associated with an elevated inflammatory CD11b+ DCs and a reduction in gut resident CD103+ DCs, it is crucial for patients with food allergy to maintain a balance between tolerogenic CD103+ DCs and inflammatory CD11b+ DCs (145). Mucosal CD103+ DCs have also been found to stimulate Foxp3+ regulatory T (Treg) cells through TGF-β1 and retinoic acid, which makes them crucial in the development of oral tolerance (146–148). Tregs control the strength of immunological responses and are crucial in controlling allergic sensitization (149).

Lung APCs, which get exposure to exogenous and endogenous AhR ligands during the asymptomatic sensitization phase of the asthmatic response, take up and process the allergen. The subsequent synthesis of Th2 cytokines and activation of B cells that develop into allergen-specific IgE-production is triggered by DCs-presentation of allergen to naïve T cells. These IgE antibodies attach to basophils and mast cells that express high IgE affinity receptors in anticipation of future allergen exposure. Both macrophages and DCs exhibit AhR expression, and new research indicate that AhR functions upstream of DC by steering the destiny of human monocytes toward DC rather than macrophages. This suggests that AhR activation may favor DC-dependent processes (150). Interestingly, mesenteric lymph node (MLN) CD103^+^ DCs elevate AhRR upon TCDD-dependent *in vivo* activation of AhR (151). However, limited information related to the AhR gene is available in association with DCs subsets (152).

AhR is extensively expressed in cDCs (101) and is crucial to control these cells. Multiple tolerogenic processes are activated in DCs by genomic and non-genomic AhR signaling, which modifies the production of cytokines essential for effector and Treg development (153). It has also been demonstrated that AhR signaling in DCs regulates the expression of metabolites with significant immunoregulatory roles. For instance, IDO, an enzyme that catalyzes the formation of the Trp-derived metabolite kynurenine, is driven by AhR and IL-6, IFN-γ, TNF-α, and IL-1β (154). It’s interesting to note that kynurenine is an AHR AhR agonist, promoting FoxP3+ Treg differentiation and reducing inflammation in various situations (114, 155). Interestingly, plant-derived indole-3-carbinol enhances CD11c+ and CD103+MHC-II+ cells in the lamina propria of mice induced with peanut allergy (100).

*In vitro* maturation of AhR-deficient LCs did not raise the expression of co-stimulatory markers CD40, CD80, and CD24a, and had a greater phagocytic capability. It’s interesting to note that AhR-deficient LC had much lower levels of tolerogenic Ido mRNA expression, and bone marrow-derived dendritic cells could not be activated by AhR (156). The generation of tolerogenic splenic CD103+ DCs and the ultimate activation of Treg cells are linked to *in vivo* AhR upregulation. Increase in Treg and the inhibition of the inflammatory response are thought to be caused by an upsurge in retinoic acid synthesis by DCs and/or an elevation in the IDO caused by AhR activation (153, 155, 157). Altogether, these studies demonstrate how AhR signaling may activate transcriptional pathways relevant to DC, making it an attractive option for medical approaches.

## AhR in macrophages to regulate allergic diseases

Macrophages are crucial in establishing adaptive immunity. These cells respond to various environmental signals and aid the development of various allergic, autoimmune, infectious, and non-infectious inflammatory diseases (158). Upon exposure to allergens, macrophages attain two primary phenotypes: the classical inflammatory M1 phenotype and the alternative anti-inflammatory M2 phenotype (159). M1 phenotype is typically activated by IFN-γ and lipopolysaccharide (LPS), which ultimately elevate the expression of inflammatory genes helpful for the clearance of intracellular pathogens. Alternatively, the M2 phenotype is activated by IL-4 and IL-13, which enhance the expression of anti-inflammatory genes involved in wound healing (160). Elevated polarization of M2 macrophage was determined in various allergic diseases, including skin allergies and allergic asthma (161–163).

Activation of M1 macrophages through LPS elevates the expression of AhR in macrophages through the NF-κB pathway (164). The loss of AhR in macrophages diminishes the IL-10 secretion in LPS-activated macrophages. Mechanistically, AhR was found to be an essential component for the secretion of IL-10 and the phosphorylation of STAT3 through Src during inflammatory macrophage-dependent immune regulation (164). Besides, both exogenous (BaP) and endogenous (FICZ) AhR ligands can enhance the Rac1 ubiquitination, which takes part in the activation of macrophages through PI3K/AKT-pathway, ensuing in a pro-inflammatory macrophage phenotype (165). Meanwhile, AhR has been known to impact the M1/M2 macrophage polarization by influencing arginine and nitric oxide production. It was determined that macrophage-specific depletion of AhR accelerates the secretion of pro-inflammatory cytokines upon stimulation with LPS, affecting the balance between M1 and M2 macrophages (166). Similarly, ablation of AhR in tumor-associated macrophages led macrophages towards a pro-inflammatory phenotype which ultimately raised the population of intra-tumoral IFNγ secreting CD8^+^ T cells (167). Another study illustrated that AhR exerts a negative role in the differentiation of monocytes and bone marrow-derived macrophages (150, 168). It was established that the activation of AhR downregulates the differentiation of macrophages by influencing the AhR-miR-142a-IRF1/HIF-1α axis to mitigate M1 macrophage polarization and expand M2 macrophage polarization (169).

In systemic lupus erythematosus, the expression of AhR is increased on the surface of macrophages susceptible to apoptotic cells, which eventually promotes the secretion of immunosuppressive cytokine IL-10; thus, limits the pathogenesis of SLE *in-vivo* (170). Meanwhile, in the cockroach extract (CRE)-induced asthma mouse model, it was estimated that macrophages polarized from mesenchymal stem cells shift pro-inflammatory M1 toward an anti-inflammatory M2 macrophage which was highly dependent on the AhR signaling. However, the AhR antagonist (CH223191) decreased the M2 polarization and boosted M1 polarization in mice challenged with cockroach allergens (96). Overall, it can be recommended that AhR is decisive in the polarization of M1 and M2 macrophages.

## AhR in B cells to regulate allergic diseases

Antigen-specific B-cell clones are activated and differentiated in response to the particular antigen when interacting with a mature naive B cell or with T cells. This happens in phases, with each stage corresponding to a modification in the genome level at the antibody loci, eventually leading to the development of memory B cells, effector cells (plasma cells), and antibody production (humoral immunity) (171). The AhR level in the various cells entangled in the pathogenesis of allergy disorders has been previously demonstrated. The IL-4 and IL-13 secretion stimulates B cells to transform them into plasma cells, producing allergen-specific IgE that binds to high-affinity IgE receptors on the mast cells. The allergen representation does the Th2 activation to naïve T cells through APCs (19). For sensitization and the emergence of the instant bronchospastic response by IgE production, the humoral response’s development in asthma is crucial.

B cells express AhR, and a prior investigation found that the AhR agonist [4-(3-chloro-phenyl)-pyrimidin-2-yl]-[4-trifluoromethyl-phenyl]-amine (VAF347)] suppresses the production of IgE by B cells (172). The AhR functions in B cells have recently been reexamined. It has been discovered to control the destiny decisions of B cells by favoring memory B cells which suppress the terminally developed antibody-secreting plasma cells (173). Additionally, the activation of the Ahr is activated by microbiota-derived butyrate, which helps in the accumulation of Il-10-secreting regulatory B cells (Bregs) (174, 175).

By inhibiting IgG and IgM production and reducing B cell development into Ig-secreting cells, TCDD inhibits humoral immune responses (176). The direct impacts of TCDD on the growth of B cells have recently been evaluated by Sulentic et al. (177). In a nutshell, TCDD interferes with B cell-dependent antibody production and their proliferation at various phases of B cell maturation and differentiation. B cells are particularly vulnerable to AhR-ligand TCDD, particularly during their activation phase, which will ultimately be low vulnerable as cells move closer to terminal differentiation. B cell activation produces a significant level of AhR.

Additionally, it’s likely that TCDD influences the transcription of various genes in B cells AhR-dependent manner and causes various biochemical modifications which can be unrelated to AhR-mediated gene transcription (177). Aside from these direct impacts on B cells, AhR stimulation can adversely impact the function and the activation activity of B cells on the Th cells and Th-secreted cytokines. As a result, we urge that AhR is highly engaged in controlling B cells, and targeting AhR can promote memory responses.

## AhR regulating T cells in allergic diseases

Airway hyperresponsiveness, eosinophil recruitment, mucus hypersecretion, and the secretion of the abovementioned mediators contribute to the initiation and progression of the allergen-specific inflammatory reaction (178, 179). The unique asthmatic reaction player from the innate lymphoid cell family (ILC) has more recently been identified as critical for allergic reactions (180). ILCs are mostly found in mucosal regions, lack antigen receptor expression, and react to cytokines in their surroundings. Innate equivalents of Th1, Th2, and Th17/Th22 cells are type 1, 2 and 3 ILCs, respectively, and their cytokine pattern is similar to that of conventional T helper cell populations. ILC2 participates in both experimental and clinical allergies by generating airway hyperresponsiveness, mucus hyperproduction and lung eosinophilia (180). ILC2 also generates IL-5 and -13 after stimulation by TSLP, IL-25, or -33. Lung ILC3 has only previously been discovered in a few experimental asthma types, particularly those that are linked to obesity (181, 182). When AhR activity is increased, the ILC2 function is suppressed, whereas ILC3 function is promoted (111, 183, 184). This suggests that AhR favors the generation of Th17-type cytokines by subtly counter-regulating ILC subsets, with the best in the gut. Besides the fact that AhR is abundantly found in ILC2 and ILC3 of the gut, no research has yet looked at how AhR ligands with allergens cause Th17-type responses, even though several studies have demonstrated this.

While the induction of Treg is considered advantageous, some kinds of asthma have a high Th17 profile that may be a factor in their severity. Th17 cells are a well-known immune regulator subgroup of CD4+ T cells that secrete the cytokines interleukin (IL)-17 (also known as IL-17A), IL-17F, and IL-22. AhR activation promotes T helper (Th)17 cell development, which worsens *in vivo* Th17 cell-mediated autoimmunity (such as EAE) (120). In a model of the ovalbumin-induced allergic disease model, it was studied that AhR regulates the differentiation of T cells by dendritic cells (93). The levels of AhR expression vary among the T cell subsets, with Th17 and Treg cells expressing the most AhR and Th1 and Th2 cells the least (185). It was undermined that indoxyl 3-sulfate (I3S) acts as AhR ligand and regulates the differentiation of Th2 cells in the ovalbumin-sensitized allergic asthma model (92). The AhR also modulates the production of cytokines by Th2 cells. 4-nonylphenol Indeno[1,2,3-cd]pyrene is a prominent air pollutant that increases the secretion of cytokines from Th2 cells in an ovalbumin-induced allergic asthma model (94, 95). AhR became a major player in the control of T cell biology in the 2000s. It has been demonstrated that AhR is increased during the maturation of Th17 cells, which increases the synthesis of IL-17 and IL-22 in mice (120, 121, 186) and IL-22 in humans (187, 188). As a result, Th17 cell production of IL-22 is reduced in AhR-deficient animals (120). However, AhR ligands may exert different effects on the Th17-mediated allergic response outcomes through FICZ as favoring and TCDD as inhibiting this profile (120, 121). During Th17 development, signal transducer and transcription 3 are activated, which causes AhR expression to increase (115). AhR also inhibits Stat5 and Stat1 signaling, which would otherwise prevent Th17 formation and create a positive feedback loop (186). It was also found that urban dust particle (SRM1649b) enhances Th17 polarization (97). Thus, AhR could contribute to the early phases of Th17 differentiation, but additional elements are necessary for the growth of fully pathogenic effector Th17 cells (189).

AhR activation has also been connected to type 1 T regulatory cells (Tr1) cells (secreting IL-10) and Foxp3 expressing Tregs (190, 191). AhR ligands like TCDD or kynurenine boost Foxp3 expression by a variety of methods, including epigenetic changes in T cells and regulation of DC, in contrast to FICZ, which encourages differentiation of Th17 cells in both non-eosinophilic asthma model and cockroach-sensitized mouse model (89, 98, 99, 155, 192). These epigenetic T cell changes, particularly DNA methylation, promote plasticity and flexibility among CD4+ T cells and enable differentiation of various subpopulations (193). Increased FOXP3 methylations in peripheral Treg cells in asthma patients have been more linked to their impaired functions in a high ambient air pollution environment than asthmatics in an environment with low air pollution (194). It is noteworthy that, other than TCDD, the non-dioxin-like AhR ligands, such as FICZ, β-NF, and 6-MCDF, exert no significant effect on the Treg in the peanut-induced allergy model (99).

Alternatively, Il-27, known as a cytokine with pleiotropic activity while performing various immune regulatory actions, has been shown to increase Tr1 cells’ AhR expression through a process mediated by Stat3 (195). The immunosuppressive cytokine IL-10 is secreted by Tr1 cells because of this activation-dependent effect on their transcriptional program. It is notable that activin-A, one of the members of the TGF-family, can stimulate the level of AhR and IRF4 transcription factors in human CD4+ T cells, resulting in the development of Tr1-like cells capable of suppressing the reactions to allergens in a mouse model (196). It is interesting to note that AhR can change mature Th17 cells into Tr1 cells that produce IL-10 (197), a mechanism evident in allergic rhinitis (198). Therefore, it is worth associating the AhR with the plasticity of Th17 cells that need stimulus to differentiate into regulatory Tr1 cells or pathogenic Th17 cells.

AhR ligands encourage the production of Foxp3 Tr1-like cells on human CD4+ T cells, which inhibit through granzyme B and produce IL-10. Additionally, AhR ligands, in the presence of TGF-β1, activate the Foxp3+ Treg, which suppresses by activating CD39, an enzyme that hydrolyzes ATP to AMP. SMAD1 is induced by TGF-β1 and AhR ligands in a mechanism that encourages Foxp3 expression by binding to the Foxp3 enhancer. Aiolos, which binds to Foxp3 and inhibits IL-2 production, is also produced to counter the AhR ligands and TGF-β1 (191, 199). AhR is also implicated in the trans-differentiation of Th17 to Tr1 since AhR agonists facilitate this process (197). AhR modulates Tr1 cell metabolism in the late phases when Hif-1 is no longer expressed (115). Interestingly, AhR and TGF-β1 form a functional axis that advances the allergic airway inflammation induced by cockroach allergens (200).

Of note, AhR is indispensable for developing Th17 and Tr1 cells along with the IL-6-dependent Th22 cells, which secrete IL-22 and function as a defense against enteropathogenic bacteria (201, 202). Meanwhile, besides the role of AhR in the differentiation of CD4+ cells, it has been evident that AhR also alters the CD8+ T cell responses in an antigen-specific manner. AhR deficiency disrupts the primary CD8+ cell responses in response to influenza virus-related infections in a cell-extrinsic way (203). Meanwhile, it has been noted that during the silencing of AhR, CD8+ T cells with antigen-specificity can display altered patterns of DNA methylation and an aberrant transcriptional profile suggesting the presence of exhausted CD8 T cells (204). The retention and/or survival of tissue-resident memory CD8 T lymphocytes in the skin is also regulated by AhR (205).

The role of AhR in γδT cell biology has also been well established. It was illustrated that IL-17-secreting innate-like γδ T cells (206, 207) increase the level of AhR, which ultimately play a pivotal role in the production of IL-22 by γδ T cells (208). Similarly, AhR signaling also plays a crucial role in maintaining the population of skin-resident Vγ3+ γδ T cells, also recognized as Dendritic Epidermal γδ T cells (DECT), which originate from thymic precursors and move to the skin during early stages of life (117, 119). Moreover, for the survival and maintenance of both CD8αα+ TCRαβ+ cells and Vγ5+ γδ T cells, intraepithelial lymphocytes in small intestinal LP, the presence of AhR ligands in the food is an essential factor (117). Together, these investigations support the notion that AhR in T cell subsets continuously interacts with the environmental cues at the mucosal level and responds to the microbial products.

## Conclusion

In conclusion, AhR controls the innate and adaptive response at a variety of levels, with consequences that are expected to vary depending on ancestry and the results of the asthmatic reaction. These results demonstrate positive and negative impacts on the allergens-specific response when using gene silencing, knockout mice, or AhR antagonists. Even though AhR was cloned more than 20 years ago, there is still much we don’t know about how it works in health and diseases. Recent research amply supports the notion that AhR plays a critical function in immune responses far beyond the mere identification of contaminants. However, the AhR crystal structure is still a mystery, and nothing is known about how nutritional, environmental, bacterial, and endogenous ligands interact with one another to influence AhR signaling.

The molecular dissection for the deep understanding of AhR signaling has focused mainly on AhR-canonical signaling, which generally represents the association of exposure to environmental pollutants with the diverse pathophysiological effects in an AhR-dependent manner. This canonical signaling is associated with the AhR heterodimerization with ARNT and XREs. It has been demonstrated that the Th1/Th2 cell balance, regulatory T cells, and dendritic cells are among the immune system cells that are impacted by the activation of the AhR in response to dioxins. Due to their involvement in the development of either tolerance or allergic sensitization, these cells, as previously mentioned, play a significant part in numerous allergies. Activating the AhR by dioxin-like substances has recently been demonstrated to decrease allergy sensitization by reducing the absolute number of precursor and effector T cells, maintaining CD4+CD25+Foxp3+ Treg cells, and altering DCs and their interaction with effector T cells in peanut allergy model (18, 209). However, in several diseases, AhR interacts with and influences numerous other genes, including Erα and NF-κB, in a non-canonical pathway. Our unpublished data shows CCl_4_, which is not a ligand for AhR and induces hepatic inflammation, also influences the accumulation of immune cells in the inflamed liver and decreases the hepatic disease severity after the knockout AhR in Tregs. Additionally, a previous study also suggests that binding of AhR with Erα exhibits higher affinity compared to ARNT, and alterations of AhR signaling significantly influence the functions of Tregs in autoimmune hepatitis (210). Thus, blocking the AhRR and Erα can potentially reestablish immune homeostasis in autoimmune diseases and other immune-mediated diseases.

Several lines of research suggest that the AhR pathway may be a valuable target for diseases, e.g., multiple sclerosis, inflammatory bowel disorders, psoriasis, cancer, and stem cell transplantation, although caution is still advised. Most likely, to get the intended outcomes, AhR activation has to be strictly regulated. For instance, proper wound healing and defense against bacterial infections are controlled by AhR-dependent IL-22 secretion from innate and adaptive cells. However, IL-22 production that is persistent and dysregulated develops into a pathogen and causes colitis and cancer (211, 212).

We have discussed that AhR is pivotal in modulating the effects on the immune response. As AhR interacts with various exogenous ligands, it serves as a potential target for numerous small molecules for the therapeutic intervention. It was reported that tapinarof and laquinimod could significantly target the AhR to treat multiple sclerosis, atopic dermatitis, and psoriasis (213–215). Similarly, metformin, which acts as an anti-diabetic drug, can also influence the activity of AhR in mast cells to treat allergic diseases (216). Similar tight regulation is probably crucial in many biological processes that AhR regulates. However, the presence of AhR in numerous tissues and cell types is challenging to treat AhR signaling pathway. Therefore, cell-specific delivery should also be considered to target this pathway. For instance, targeted delivery of chemically modified Foxp3 mRNA to sites of inflammation in the house dust mite-induced allergic asthma served as a safe and efficient therapeutic tool by regulating T cell immune responses (217). Overall, it appears that AhR pathway is activated through numerous signals from several sources to ensure that the host responds accurately and adapts to ongoing environmental changes and allergens, a step crucial to adaptation.

## Author contributions

FR, FP, WP outlined the manuscript,FR drafted it. FP and PW reviewed and improved the rigorousness of the manuscript. FP and PW supervised the study. All authors contributed to the article and approved the submitted version.
